# Characterization of McCIPK6, a mitochondrial protein, positively regulating cold stress tolerance in bitter gourd (*Momordica charantia* L.)

**DOI:** 10.3389/fpls.2026.1856408

**Published:** 2026-05-21

**Authors:** Xuetong Yang, Kai Wang, Yuanyuan Xie, Feng Guan, Bo Shi, Xinjian Wan

**Affiliations:** 1 Institute of Vegetables and Flowers, Jiangxi Academy of Agricultural Sciences, Nanchang, China; 2 Jiangxi Key Laboratory of Horticultural Crops (Fruit, Vegetable & Tea) Breeding, Jiangxi Academy of Agricultural Sciences, Nanchang, China; 3 Jiangxi Engineering Research Center of Vegetable Molecular Breeding, Jiangxi Academy of Agricultural Sciences, Nanchang, China

**Keywords:** CBF signaling pathway, CIPK gene, cold stress, McCBL1, *Momordica charantia*, reactive oxygen species

## Abstract

As an economically important vegetable species, bitter gourd *(Momordica charantia* L.) is widely cultivated around the globe. However, its production is frequently threatened by late spring coldness, which severely impairs seedling establishment, delays flowering and fruit set, and reduces both yield and quality. Unraveling mechanisms of cold resistance in bitter gourd is therefore critical for breeding resilient cultivars and expanding its planting range. Here, we found a cold-inducible *CIPK* family gene, *McCIPK6*, from bitter gourd via transcriptome screening, and systematically characterized its function in cold stress responses using phenotypic, physiological, and molecular approaches. Expression analysis revealed that *McCIPK6* was rapidly and strongly induced in the cold-tolerant genotype ‘0208’ (R) under low temperature, whereas the cold-sensitive line ‘2206’ (S) showed a delayed and dysregulated expression pattern. Mitochondrial localization analysis revealed that McCIPK6 is a mitochondria−targeted CIPK kinase. Heterologous overexpression of *McCIPK6* in Arabidopsis significantly enhanced cold tolerance by simultaneously promoting mitochondrial ROS scavenging and activating the *CBF*‐dependent cold signaling pathway. These effects were reflected by improved survival rates, reduced MDA accumulation, lower ROS levels, higher antioxidant enzyme activities (SOD, POD, CAT), and upregulated expression of *CBF* regulation genes as well as antioxidant genes. Further verification via yeast two-hybrid and luciferase complementation experiments revealed that McCIPK6 physically interacts with McCBL1, a core calcium sensor in bitter gourd. Collectively, our data establish that *McCIPK6* functions as a positive regulator of cold tolerance by regulating ROS scavenging and the *CBF*-dependent cold signaling cascade, likely through forming a functional complex with McCBL1. This study reveals a novel regulatory module underlying cold adaptation in bitter gourd and offers a valuable candidate gene for the genetic enhancement of cold resistance in cucurbit crops.

## 1 Introduction

Bitter gourd, scientifically named *Momordica charantia* L., is classified as an annual climbing herb of the Momordica genus in the Cucurbitaceae family. As an economically important vegetable crop, bitter gourd exhibits high nutritional value, strong disease and insect resistance, antimicrobial activity, and multiple health−promoting characteristics. However, late spring coldness severely hinders early-spring cultivation, leading to significant yield losses due to reduced seedling survival, delayed flowering, and malformed fruits. Exploring the mechanisms underlying low−temperature tolerance and breeding cold−tolerant varieties are the most effective strategies to alleviate cold injury. Studies on the low−temperature stress response of bitter gourd will support the breeding of cold−adapted cultivars, enabling earlier transplanting and marketing in early spring, which is critical for ensuring food security. Furthermore, research on cold tolerance in bitter gourd provides a molecular basis for genetic improvement, expands cultivation to colder regions, reduces transportation costs, improves land use efficiency, and enhances overall agricultural productivity. Therefore, investigations into the molecular basis of cold stress tolerance in bitter gourd are urgently needed, and basic research in this field has important theoretical and practical significance.

In plants, calcium ions (Ca²^+^) as key signaling messengers to cope with numerous abiotic stresses, including low temperature, water deficit, and salt stress (Hepler, 2005). Ca²^+^-sensing proteins exert crucial functions during the transduction of calcium signals. In higher plants, four major families of Ca²^+^ sensors have been characterized, namely calmodulins (CaMs), calmodulin-like proteins (CMLs), Ca²^+^-dependent protein kinases (CDPKs), and calcineurin B-like proteins (CBLs) (Chen et al., 2024). The CBL-CIPK signaling network is formed through the recognition of Ca²^+^ signals by CBL proteins and their subsequent interaction with CIPKs, exerting key functions in plant development and tolerance to various stresses (Tang et al., 2020). Genomic evidence indicates that the CBL-CIPK network is widely conserved in plants, and the expansion of *CBL* and *CIPK* gene families resulted from local and whole-genome duplication events, which may have co-evolved with plant adaptation to diverse environmental stresses. In the model plant *Arabidopsis thaliana*, 10 *CBLs* and 26 *CIPKs* have been identified. Each CBL specifically interacts with distinct CIPKs, and each CIPK may associate with one or more CBLs, forming specific and flexible functional complexes. Such specific and overlapping interactions contribute to signaling specificity and functional coordination *in vivo* (Kolukisaoglu et al., 2004; Ma et al., 2020). Accumulating studies demonstrate that the CBL-CIPK network is widely involved in responses to various abiotic stresses, including salinity, osmotic stress, drought, low temperature, ABA, low potassium, nitrate, and hypoxia. The CBL-CIPK pathway also crosstalk with other key signaling modules, such as CDPK, AMPK, SOS, and ROS pathways. Modulation of the CBL-CIPK network significantly improves plant tolerance to multiple abiotic stresses, highlighting its great potential for crop stress-resistance breeding (de la Torre et al., 2013; Kaya et al., 2024; Manik et al., 2015). *CIPK6* is prominently activated at the transcriptional level under abiotic stress conditions. Overexpression of *BnCIPK6* from *Brassica napus* in Arabidopsis enhances salt tolerance and ABA sensitivity (Chen et al., 2012, Chen et al., 2013). AtCIPK6 improves drought and salt tolerance by regulating the calcium-binding protein AtCBP (Tsou et al., 2012). In Nymphaea, CIPK6 interacts with SnRK1 and promotes its accumulation; activated SnRK1 degrades NCED2, represses ABA biosynthesis, and impairs the ABA-mediated ROS-scavenging system, which contributes to the subgenome hybridization barrier (Zhou et al., 2025). In Arabidopsis, CIPK6 interacts with CBL4 to jointly control the activity and plasma membrane targeting of the AKT2 potassium channel (Held et al., 2011). In cotton, GhCIPK6 is targeted to the tonoplast by GhCBL2 and interacts with the sugar transporter GhTST2 to promote sugar accumulation. Silencing *GhCIPK6* reduces sugar accumulation, whereas overexpression elevates it, and this function is conserved in transgenic Arabidopsis (Deng et al., 2020). Although *CIPK6* has been reported to involved in abiotic stress responses, whether *CIPK6* regulates cold tolerance by targeting mitochondria to modulate ROS homeostasis and coordinate the *CBF* pathway remains largely unknown, especially in thermophilic horticultural crops such as bitter gourd. This study is the first to characterize a mitochondria−localized McCIPK6 that confers cold tolerance through a dual mechanism integrating mitochondrial ROS scavenging and *CBF* signaling, which fills the knowledge gap of CBL–CIPK function in bitter gourd cold adaptation.

Our previous transcriptome data demonstrated that *McCIPK6* was markedly induced by cold stress, suggesting its potential function in cold tolerance. In this study, we systematically investigated the expression pattern, subcellular localization, and biological function of McCIPK6 in bitter gourd using cold-tolerant (R) and cold-sensitive (S) genotypes. We further explored its molecular mechanism by analyzing antioxidant capacity, cold-related gene expression, and protein-protein interactions. This research aims to uncover the regulatory mechanism of *McCIPK6* in bitter gourd cold tolerance, offering a theoretical framework to facilitate the genetic breeding of cold-tolerant vegetable crops.

## 2 Materials and methods

### 2.1 Plant materials and cultivation condition

In our study, two inbred lines of bitter gourd with contrasting cold tolerance were employed, including the ‘0208’ (cold-resistant material, R) and the line ‘2206’ (cold-sensitive material, S), which were supplied by Jiangxi Academy of Agricultural Sciences. Seeds were soaked in distilled water for 24 h, followed by germination at 32 °C with 80% relative humidity for 48 h. After germination, the seeds were sown separately in plug trays and cultured in a growth chamber under the conditions as follows, 14 h light/10 h dark photoperiod, day/night temperature of 30 °C/28 °C, and light intensity of 10000 lx. Seedlings were subjected to 5 °C low−temperature stress at the four leaf one heart stage. The fully expanded functional leaves were collected at 0, 6, 12, and 24 h after treatment for gene expression analysis.

*Arabidopsis thaliana* (Col-0) seeds were sown directly into the culture substrate with a ratio of nutrient soil: vermiculite: perlite = 3:1:1. The seeds were stratified at 4 °C for 3 days and then placed in growth chamber maintained at 22 °C for culture. The culture conditions were set as follows, 16 h light/8 h dark photoperiod, day/night temperature of 22 °C/20 °C, and light intensity of 20000 lx. After the Arabidopsis seedlings developed two true leaves, healthy seedlings with uniform growth were selected for transplanting. For cold tolerance assay in Arabidopsis, three-week-old wild-type (WT) and transgenic seedlings were exposed to −14 °C for 1.5 h, then transferred to 4 °C for 16 h, and finally allowed to recover at 22 °C for 2 days. Plant phenotypes were documented, and survival rates were determined.

### 2.2 RNA extraction and RT-qPCR analysis

Leaf samples were used for total RNA extraction with TRIZOL reagent, and a reverse transcription kit was employed to synthesize first-strand cDNA. RT-qPCR experiments were conducted using a fluorescent quantitative PCR kit on the QuantStudio™ 7 Flex system (Applied Biosystems, USA). The 2^-^^ΔΔCT^ method was applied to determine relative gene expression levels, and all reactions were performed in triplicate. **Supplementary Table 1** shows the primers used in this research.

### 2.3 GUS histochemical staining

The promoter fragment of McCIPK6 was amplified and inserted into the pCAMBIA1391 vector to generate the proMcCIPK6::GUS fusion construct. The recombinant vector was transformed into wild-type *Arabidopsis thaliana* by the floral dip method to obtain positive transgenic plants. Subsequent to cold treatment, the samples were incubated at 37 °C in GUS staining solution for 24 hours, and decolorization was conducted with 70% ethanol afterward. GUS signals were observed and recorded using an Olympus SZX10 stereomicroscope. The primers utilized are listed in **Supplementary Table 1**.

### 2.4 Subcellular localization

The coding sequence of McCIPK6 without the stop codon was cloned into the PYBA1132 GFP vector to generate the 35S::McCIPK6 GFP fusion construct. The recombinant plasmid and empty vector were transformed into Arabidopsis protoplasts. Mitochondrial marker RFP was co-transformed as a localization control. Fluorescence signals were observed and captured using an IX83 FV1200 laser confocal scanning microscope (Olympus, Japan). The primers utilized are listed in **Supplementary Table 1**.

### 2.5 Generation of *McCIPK6*-overexpressing *Arabidopsis*

To generate *McCIPK6*-overexpressing Arabidopsis plants, the full-length coding DNA sequence (CDS) of *McCIPK6* was amplified and inserted into the pBI121 overexpression vector, in which target gene expression was driven by the constitutive CaMV 35S promoter. The successfully constructed recombinant vector was subsequently introduced into *Agrobacterium tumefaciens* strain GV3101 through heat-shock transformation. Wild-type *Arabidopsis thaliana* plants were then transformed with the positive recombinant *Agrobacterium* strain using the floral dip method. After transformation, transgenic seedlings were screened and selected on half-strength Murashige and Skoog (1/2 MS) solid medium supplemented with 50 μg/mL kanamycin. Through successive resistance screening and identification, three independent homozygous overexpression lines (designated OE-1, OE-2, and OE-3) at the T_3_ generation were obtained for subsequent phenotypic and functional analyses. The primers utilized are listed in **Supplementary Table 1**.

### 2.6 DAB and NBT staining

To histochemically detect hydrogen peroxide (H_2_O_2_) and superoxide anion (O_2_^-^), leaf samples were incubated with 3,3′-diaminobenzidine (DAB) and nitroblue tetrazolium (NBT) staining solutions in darkness at room temperature. Following staining, chlorophyll was eliminated via boiling in 95% ethanol. The staining results were observed and documented using a stereomicroscope.

### 2.7 Yeast two-hybrid assay

The coding sequence of McCIPK6 was constructed into the pGBKT7 vector as bait, and McCBL1 was cloned into the pGADT7 vector as prey. Both recombinant plasmids were co-introduced into yeast strain Y2HGold. Autoactivation activity was tested on selective media, and 0.25 mM 3-AT was used to suppress background activation. Protein–protein interactions were determined according to yeast growth and blue color development on SD/−Trp/−Leu/−His/−Ade + X α gal plates. The primers utilized are listed in **Supplementary Table 1**.

### 2.8 Luciferase complementation imaging assay

The full-length CDS sequences of McCIPK6 and McCBL1 (excluding stop codons) were separately fused into the pCAMBIA1300-nLUC and pCAMBIA1300-cLUC vectors. The resulting recombinant plasmids were transformed into GV3101 and then co-infiltrated into tobacco leaves. After 48 h, 1 mM luciferin substrate was injected into the infiltrated regions and kept in darkness for 5 min. Fluorescence imaging (LCI) was performed using a PlantView100 multispectral dynamic fluorescence imaging system. For quantitative analysis, the firefly luciferase activity (RLU) was measured using a luciferase assay kit. The primers utilized are listed in **Supplementary Table 1**.

### 2.9 Statistical analysis

Each experiment was conducted with three biological replicates. Values are presented as mean ± standard error (SE). Statistical significance was determined using independent-samples *t*-test and one-way ANOVA followed by Duncan’s multiple range test (* *P* < 0.05, ** *P* < 0.01).

## 3 Results

### 3.1 Characterization of *McCIPK6* in bitter gourd

**Figure 1:**
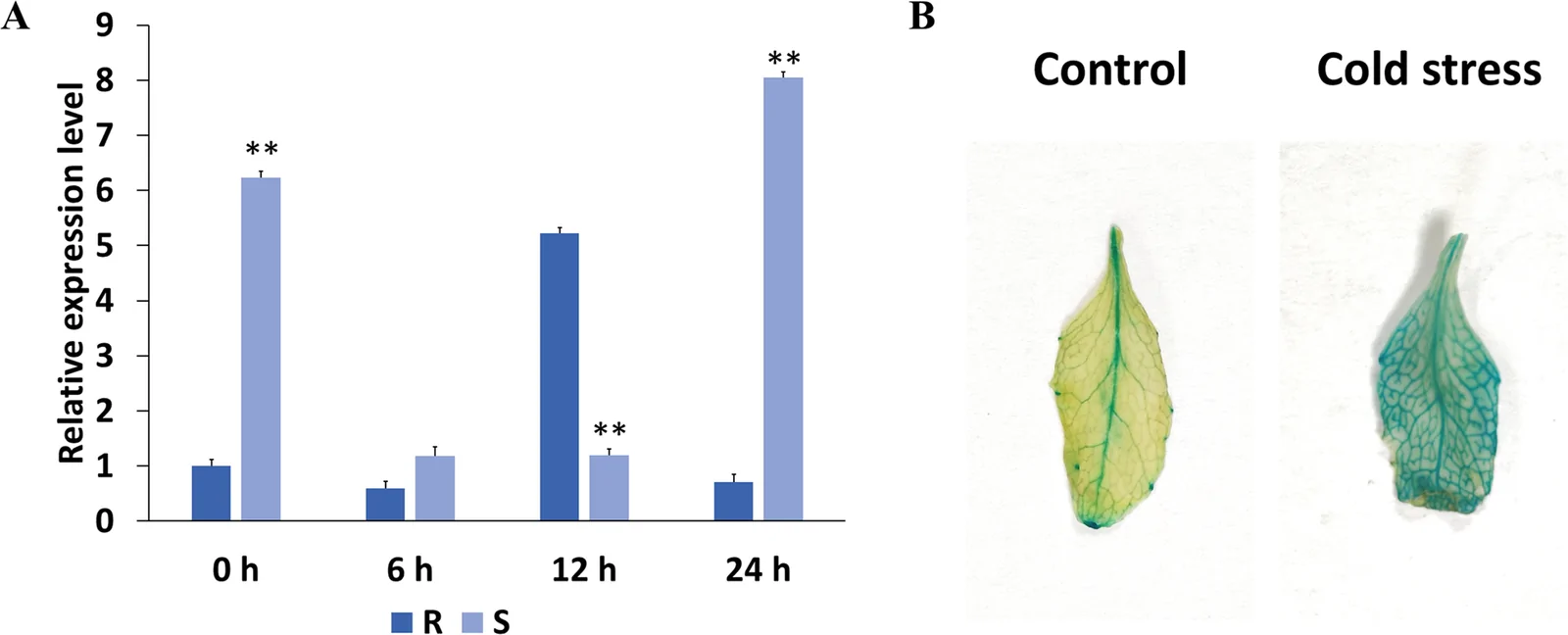
Expression analysis and histochemical staining of *McCIPK6* in cold-tolerant (R) and cold-sensitive (S) bitter gourd under low-temperature stress. **(A)** relative expression levels of *McCIPK6* in R and S materials at 0 h (control), 6 h, 12 h, and 24 h of low-temperature treatment. Blue columns represent the cold-tolerant line (R), light blue columns represent the cold-sensitive line (S). Error bars indicate the mean ± standard error (n = 3). **P < 0.01 (independent samples *t*-test, significant difference between R and S). **(B)** histochemical staining of bitter gourd leaves under control and cold stress conditions, showing the tissue-specific expression pattern of *McCIPK6*.

Based on our previous transcriptome profiling (Yang et al., 2025), *McCIPK6* was identified as a cold-responsive gene, showing significant up-regulation at 12 hours post-cold treatment. Quantitative real-time PCR (RT-qPCR) assays demonstrated distinct expression dynamics of *McCIPK6* in R and S genotypes under cold stress. At 0 h (control condition), the basal expression level of *McCIPK6* was higher in S than in R. Following 6 h of cold stress, both genotypes displayed reduced expression, with S still maintaining a slightly higher level than R. Notably, *McCIPK6* expression in R rapidly peaked to a significantly higher level at 12 h, whereas that in S remained low. By 24 h, expression in R returned to near baseline, while that in S surged to its maximum level, far exceeding its initial value (**Figure 1A**). This expression pattern suggested that R responded to cold stress promptly and adaptively, whereas S exhibits a delayed and excessive reactive response, indicative of passive stress damage rather than active regulation.

To examine the tissue-specific expression characteristics of *McCIPK6*, we generated a *McCIPK6* promoter-driven GUS fusion vector and introduced it into Arabidopsis through the floral dip approach. Under non-stress conditions, weak GUS activity was primarily detected in the leaf veins, suggesting a low basal level of *McCIPK6* expression. In contrast, cold stress markedly enhanced GUS staining intensity, with strong blue signals spreading throughout the leaf lamina (**Figure 1B**). These results indicated that *McCIPK6* was predominantly expressed in leaf veins under normal growth conditions and is significantly activated by cold treatment, implying its critical role in mediating cold response signaling pathways.

### 3.2 Subcellular distribution of McCIPK6

**Figure 2:**
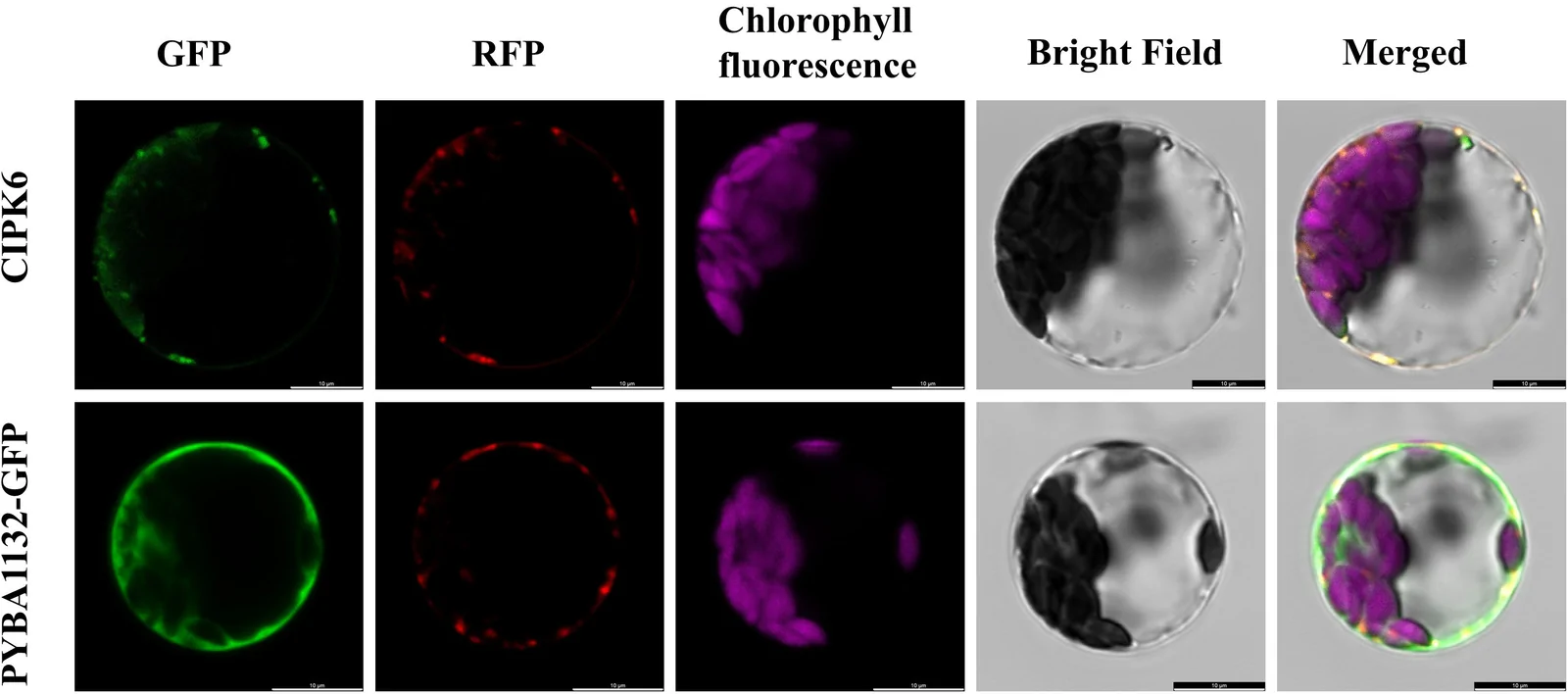
Subcellular localization of McCIPK6 in *Arabidopsis* protoplasts. The McCIPK6-GFP fusion construct and the empty vector PYBA1132-GFP were transiently expressed in Arabidopsis protoplasts. From left to right: GFP channel (green, showing fusion protein localization), RFP channel (red, mitochondrial marker), chlorophyll autofluorescence (purple, chloroplast marker), bright-field image, and merged image. Scale bar = 10 μm.

To investigate where McCIPK6 is localized within cells, we transiently expressed the recombinant vector 35S::McCIPK6-GFP in Arabidopsis protoplasts, using the empty pYBA1132-GFP vector as a negative control. Observation via confocal laser scanning microscopy indicated that the control GFP fluorescence formed a clear ring-like pattern at the cell periphery, consistent with the cytoplasmic membrane. In contrast, the McCIPK6-GFP fusion protein was distributed as discrete fluorescent dots in the cytoplasm. Co-localization analysis with a mitochondrial marker (RFP) showed almost complete overlap of McCIPK6-GFP with the RFP signal, whereas no co-localization was observed with chlorophyll autofluorescence. These results conclusively demonstrated that McCIPK6 was specifically localized to mitochondria in plant cells (**Figure 2**).

### 3.3 McCIPK6 promotes cold tolerance in *Arabidopsis* by strengthening antioxidant defense capacity

**Figure 3:**
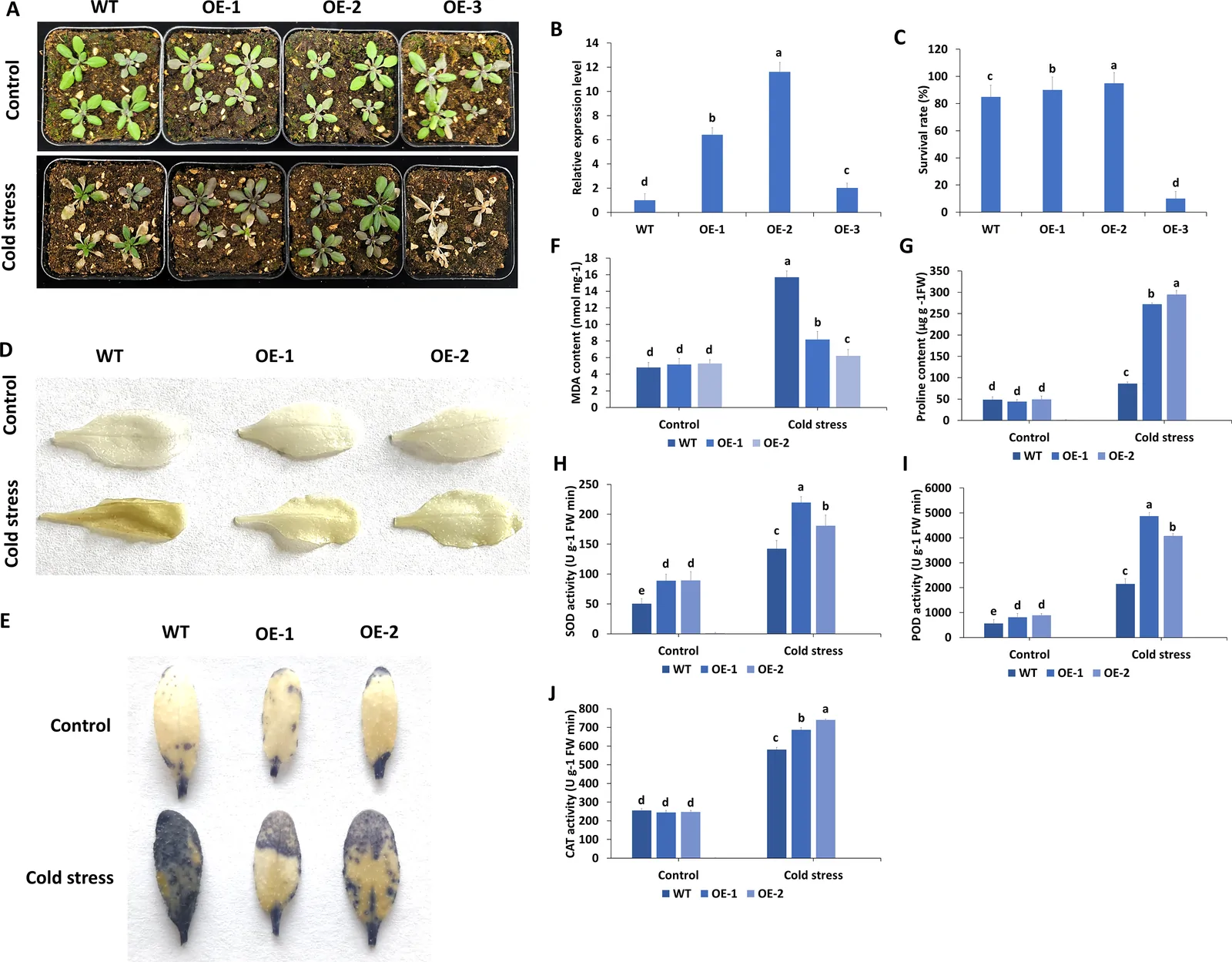
Overexpression of *McCIPK6* enhances cold tolerance in *Arabidopsis thaliana*. **(A)** Phenotypes of wild-type (WT) and *McCIPK6*-overexpressing lines (OE-1, OE-2, OE-3) under normal growth conditions and cold stress conditions (−14 °C for 1.5 h, 4 °C for 16 h, then recovery at 22 °C for 2 d). **(B)** Relative expression levels of *McCIPK6* in WT and OE lines. **(C)** Survival rates of WT and OE lines after cold stress treatment. **(D)** Representative leaf phenotypes of WT, OE-1, and OE-2 under control and cold stress conditions. **(E)** NBT staining of leaves from WT, OE-1, and OE-2 under control and cold stress conditions, showing *in situ* accumulation of superoxide anion (O_2_^-^). **(F–J)** Physiological indices of WT, OE-1, and OE-2 under control and cold stress conditions: **(F)** malondialdehyde (MDA) content; **(G)** proline content; **(H)** superoxide dismutase (SOD) activity; **(I)** peroxidase (POD) activity; **(J)** catalase (CAT) activity. The presented data are the means ± standard deviations of three independent experiments. Different letters above the bars indicate significant differences according to ANOVA with Duncan’s multiple range tests, *P* < 0.05.

To functionally characterize *McCIPK6*, we generated three independent Arabidopsis lines overexpressing *McCIPK6* (OE-1, OE-2, OE-3) and challenged them with cold stress (−14 °C for 1.5 h, then 4 °C for 16 h, with recovery at 22 °C for 2 days). We observed no obvious phenotypic variations between WT and transgenic lines under normal growth conditions. Nevertheless, after cold stress exposure, WT plants displayed severe wilting and leaf chlorosis, while OE-1 and OE-2 lines maintained plump leaves and relatively vigorous growth. Further analysis revealed that OE-3 had a much lower expression level of *McCIPK6* (likely due to a T-DNA insertion position effect), which correlated with its lack of cold tolerance. The survival rates of WT, OE-1, OE-2, and OE-3 were 85%, 90%, 95%, and 10%, respectively (**Figures 3A–C**). These results confirmed that *McCIPK6* positively regulated cold tolerance in Arabidopsis. We therefore used OE-1 and OE-2 for subsequent mechanism analysis. Histochemical staining with DAB and NBT was used to detect the content of hydrogen peroxide (H_2_O_2_) and superoxide anion (O_2_^-^), respectively. ROS accumulation showed no significant discrepancy between WT and overexpression lines under normal circumstances. Upon cold stress, WT leaves showed extensive dark brown (DAB) and blue-purple (NBT) staining, indicating massive ROS accumulation. In contrast, OE lines exhibited significantly weaker staining intensities and smaller stained areas (**Figures 3D, E**), demonstrating that overexpression of *McCIPK6* effectively suppresses cold-induced ROS accumulation. Physiological assays revealed that under cold stress, OE lines had significantly lower MDA content, higher Pro content, and enhanced activities of SOD, POD, and CAT compared to WT (**Figures 3F–J**). Collectively, these phenotypic and physiological data indicated that *McCIPK6* overexpression reduced ROS accumulation by enhancing antioxidant system activity, thereby alleviating oxidative damage and improving cold tolerance in transgenic Arabidopsis.

### 3.4 *McCIPK6* regulates stress-antioxidant gene expression in *Arabidopsis*

**Figure 4:**
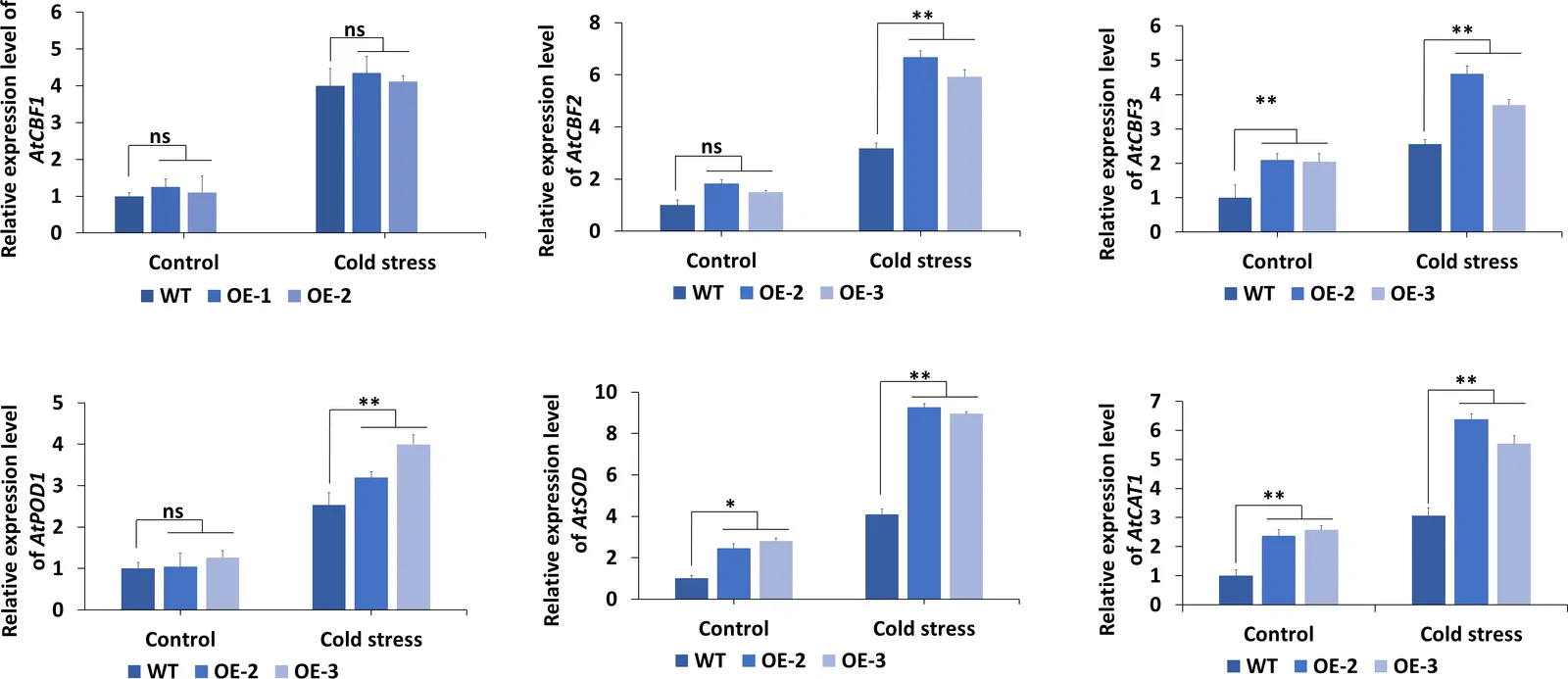
Expression analysis of cold-responsive and antioxidant-related genes in *McCIPK6*-overexpressing *Arabidopsis* lines under cold stress. The relative expression levels of cold-responsive marker genes (*AtCBF1*, *AtCBF2*, *AtCBF3*) and antioxidant-related genes (*AtPOD1*, *AtSOD*, *AtCAT1*) were detected by RT−qPCR in wild-type (WT) and *McCIPK6*-overexpressing lines (OE−1, OE−2, OE−3) under normal growth conditions (Control) and after 4 °C cold stress treatment, respectively. Error bars indicate mean ± standard error (n=3). ns indicates no significant difference, * and ** represent significant differences at *P* < 0.05 and *P* < 0.01 levels, respectively (independent-samples *t*-test, OE lines vs. WT).

To uncover the molecular mechanism underlying *McCIPK6*-mediated cold tolerance, we quantified the transcript levels of cold-responsive marker genes (*AtCBF1*, *AtCBF2*, *AtCBF3*) and antioxidant-associated genes (*AtPOD1*, *AtSOD*, *AtCAT1*) via RT-qPCR analysis. Under normal growth conditions, OE lines exhibited significantly higher transcript abundance of *AtCBF3*, *AtSOD*, and *AtCAT1* compared with WT, whereas *AtCBF1*, *AtCBF2*, and *AtPOD1* displayed no remarkable differences. Upon cold stress exposure (4 °C), the expression levels of *AtCBF2*, *AtCBF3*, *AtPOD1*, *AtSOD*, and *AtCAT1* in OE lines were further induced and markedly higher than those in WT plants (**Figure 4**). Collectively, these findings indicated that *McCIPK6* functioned as a key positive regulator in the cold stress response network, improving cold tolerance via two synergistic regulatory pathways. On one hand, it modulated the expression of *AtCBF* family genes to activate the cold signaling cascade, thus enhancing the perception and transmission of cold signals in plants. On the other hand, it upregulated the transcription of antioxidant-related genes to boost reactive oxygen species (ROS) scavenging ability and mitigate cold-triggered oxidative damage.

### 3.5 McCIPK6 interacts with McCBL1

**Figure 5:**
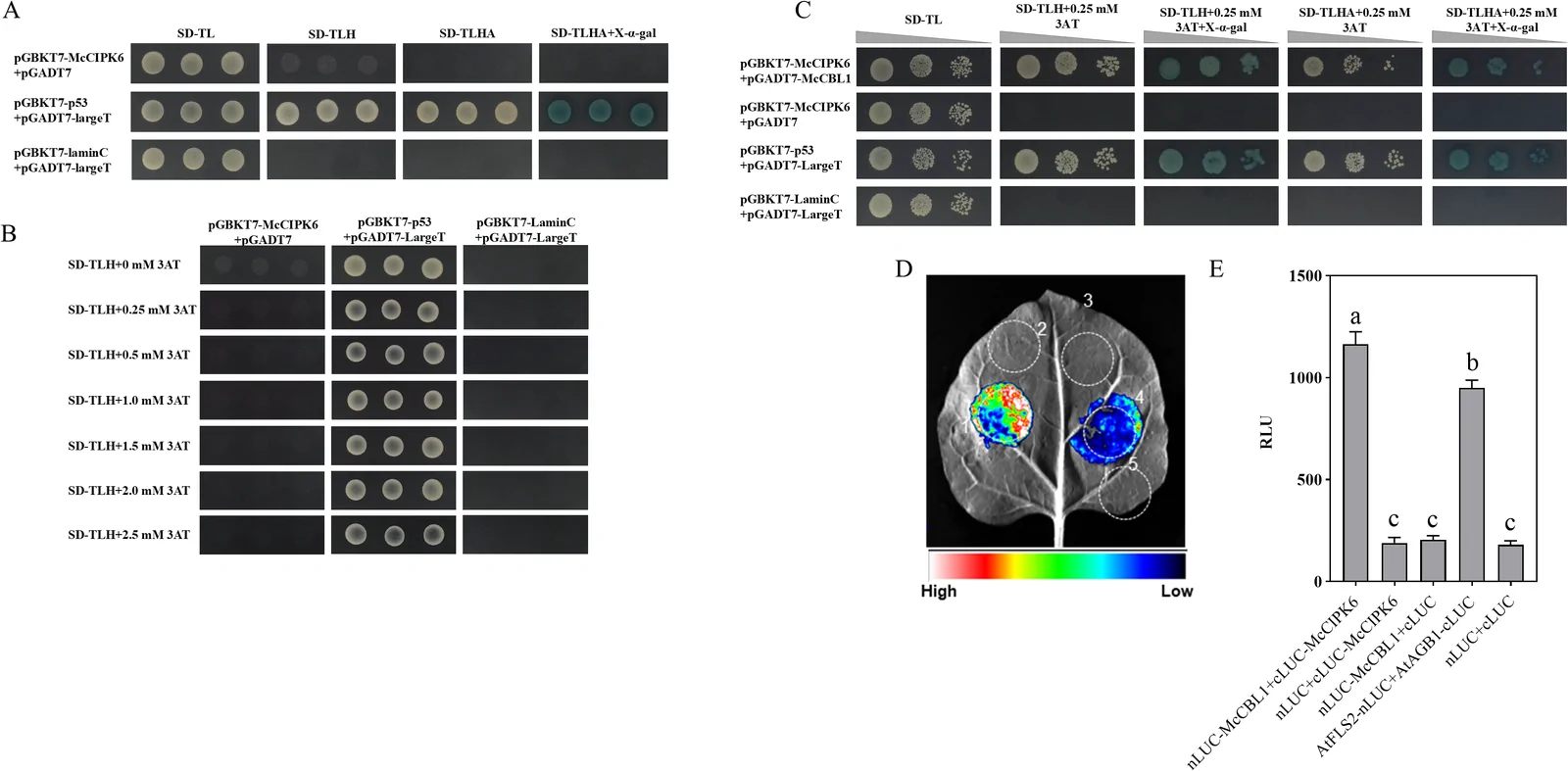
Validation of the interaction between McCIPK6 and its interacting proteins by Y2H and LCA assays. **(A)** autoactivation assay of McCIPK6. **(B)** suppression of autoactivation by 3AT treatment. **(C)** yeast two-hybrid analysis of the interaction between McCIPK6 and McCBL1. **(D, E)** luciferase complementation imaging (LCI) assay of the interaction between McCIPK6 and McCBL1 in tobacco. pGBKT7-p53 + pGADT7-largeT was used as the positive control, while pGBKT7-laminC + pGADT7-largeT served as the negative control. The presented data are the means ± standard deviations of three independent experiments. Different letters above the bars indicate significant differences according to ANOVA with Duncan’s multiple range tests, *P* < 0.05.

The interacting proteins of McCIPK6 were predicted using the online STRING database (https://cn.string-db.org/cgi/input?sessionId=baxg4Qu5oPID&input_page_show_search=on), and the authenticity of the interaction was verified by Y2H and LCI assay (**Figure 5**). McCIPK6 showed autoactivation, which was fully suppressed by 0.25 mM 3-AT (**Figures 5A, B**). Y2H assays demonstrated that the experimental group (pGBKT7-McCIPK6 + pGADT7-McCBL1) exhibited normal growth on SD-TL, SD-TLH supplemented with 0.25 mM 3-AT, and SD-TLHA selective media containing 0.25 mM 3-AT and X-α-gal and developed a blue color on the SD-TLHA + 0.25 mM 3-AT + X-α-gal plate. These results indicated that McCBL1 interacts with McCIPK6 (**Figure 5C**). The luciferase assay showed that both the positive control (AtFLS2-nLUC + AtAGB1-cLUC) and the experimental group (pCAMBIA1300-nLUC-McCIPK6 + pCAMBIA1300-cLUC-McCBL1) exhibited strong fluorescence signals, whereas no fluorescence was detected in the other negative controls, confirming the interaction between McCBL1 and McCIPK6. In addition, microplate reader detection revealed that the luciferase activity of the experimental group pCAMBIA1300-nLUC-McCBL1 + pCAMBIA1300-cLUC-McCIPK6 was significantly higher than that of the negative controls. Quantitative data further verified that McCBL1 interacts with McCIPK6 (**Figures 5D, E**).

## 4 Discussion

### 4.1 Conservation and functional characteristics of *CIPK* genes in plant cold stress response

The *CIPK* gene family has been widely reported to play critical roles in plant cold stress responses. Although the functions of *CIPKs* are partially conserved among plant species, they also show obvious species-specific characteristics. In pineapple (*Ananas comosus*), the *AcCIPK5* gene is homologous to *Arabidopsis thaliana CIPK12*. Heterologous constitutive expression of *AcCIPK5* in Arabidopsis can significantly enhance the plant’s tolerance to osmotic stress, drought, salt stress, and cold stress. This enhancement is realized by regulating the abscisic acid (ABA) signaling pathway and maintaining the homeostasis of reactive oxygen species (ROS), which confirmed that the stress-resistant function of *CIPK* genes was conserved among diverse plant species (Aslam et al., 2022). This was consistent with our finding that heterologous overexpression of *McCIPK6* in Arabidopsis enhanced cold tolerance, further confirming the conserved role of *CIPK* genes in plant cold responses. In pepper (*Capsicum annuum*), mutation of *CaCIPK13* obviously weakened cold tolerance, as demonstrated by elevated contents of MDA and H_2_O_2_, higher electrolyte leakage, as well as distinctly reduced activities of antioxidant enzymes (CAT, POD, SOD) and anthocyanin accumulation. In contrast, constitutive overexpression of *CaCIPK13* in tomato (*Solanum lycopersicum*) notably enhanced cold tolerance, accompanied by increased anthocyanin accumulation and improved antioxidant enzyme activities. Importantly, the protein–protein interaction between CaCIPK13 and CaCBL1/6/7/8 was found to be strictly dependent on Ca²^+^ signaling, highlighting the conserved Ca²^+^-sensing mechanism involved in CBL-CIPK-mediated stress regulation (Ma et al., 2022). In the present study, Arabidopsis overexpressing *McCIPK6* exhibited lower MDA content, higher proline content, and enhanced activities of SOD, POD, and CAT under cold stress. Moreover, McCIPK6 physically interacted with McCBL1, similar to the mechanism reported for CaCIPK13. These results indicated that the CBL-CIPK module was functionally conserved in plant cold tolerance regulation and also implied that McCIPK6 may regulate the cold stress response of bitter gourd by binding to McCBL1 and relying on Ca²^+^ signaling. In citrus, heterologous expression of *CuCIPK16* markedly improved cold resistance in transgenic Arabidopsis, accompanied by elevated POD activity and decreased MDA accumulation (Xiao et al., 2022). Overexpression of *OsCIPK03* in rice can improve plant cold tolerance by increasing proline and soluble sugar contents (Xiang et al., 2007). Similarly, overexpression of *LlaCIPK* (homologous to Arabidopsis *CIPK15*) in tobacco can enhance cold tolerance by increasing proline levels and cell membrane stability (Aslam et al., 2019). Together, these studies demonstrated that *CIPK* genes can enhanced plant cold tolerance by regulating pathways such as the antioxidant system and the accumulation of osmotic adjustment substances. This is consistent with the mechanism identified in our study, where *McCIPK6* modulates the cold tolerance of *Arabidopsis thaliana* by increasing antioxidant enzyme activity and reducing ROS accumulation. These results further confirmed that the regulatory pathway by which *CIPK* genes regulated plant cold tolerance through the antioxidant system and osmotic adjustment was widely conserved across plant species. In addition, virus-induced gene silencing (VIGS) experiments have revealed that tomato *SlCIPK1* and *SlCIPK8* silenced lines are more sensitive to low-temperature stress (Zhang et al., 2019), indicating that these *CIPK* genes are essential for plant cold tolerance. Tea tree *CsCIPK11* can enhance the stability and enzyme activity of the cold tolerance-related gene *CsGSTU23* through phosphorylation, thereby improving the cold tolerance of tea trees (Di et al., 2024). *Brachypodium distachyon BdCIPK31* can be induced by low temperature, and its overexpression enhances tobacco cold tolerance by regulating oxidative stress and osmotic stress (Luo et al., 2018). Similarly, overexpression of *TaCIPK14* significantly improves tobacco cold tolerance (Deng et al., 2013). In the present study, heterologous overexpression experiments in *Arabidopsis thaliana* clarified the cold tolerance function of *McCIPK6*. There was no significant difference in growth phenotype between *McCIPK6* overexpression lines (OE-1, OE-2) and the WT under non-stress conditions. After cold stress, WT plants showed severe wilting and yellowing, while OE-1 and OE-2 lines maintained a relatively robust growth state, with significantly higher survival rates than WT, indicating that the cold tolerance regulatory function of *McCIPK6* depends on its expression level. Further physiological and molecular mechanism analysis showed that *McCIPK6* overexpression significantly inhibited cold-induced accumulation of H_2_O_2_ and O_2_^-^, reduced MDA content, increased proline accumulation, and enhanced the activities of antioxidant enzymes such as SOD, POD, and CAT. This suggests that *McCIPK6* can enhance plant cold tolerance by improving the function of the antioxidant system, reducing ROS accumulation, and alleviating membrane lipid peroxidation damage. RT-qPCR results indicated that *McCIPK6* overexpression significantly upregulated the expression of *CBF* family genes (*AtCBF2*, *AtCBF3*) and antioxidant-related genes (*AtPOD1*, *AtSOD*, *AtCAT1*) in *Arabidopsis thaliana*. Since *AtCBF* family genes are core regulators in the plant cold signaling pathway, capable of activating the expression of a series of downstream cold tolerance genes, we speculate that *McCIPK6* regulates plant cold tolerance through the synergistic effect of activating the *CBF* signaling pathway and enhancing the antioxidant system.

### 4.2 Functional identification and regulatory mechanism of *McCIPK6* in bitter gourd cold stress tolerance

**Figure 6:**
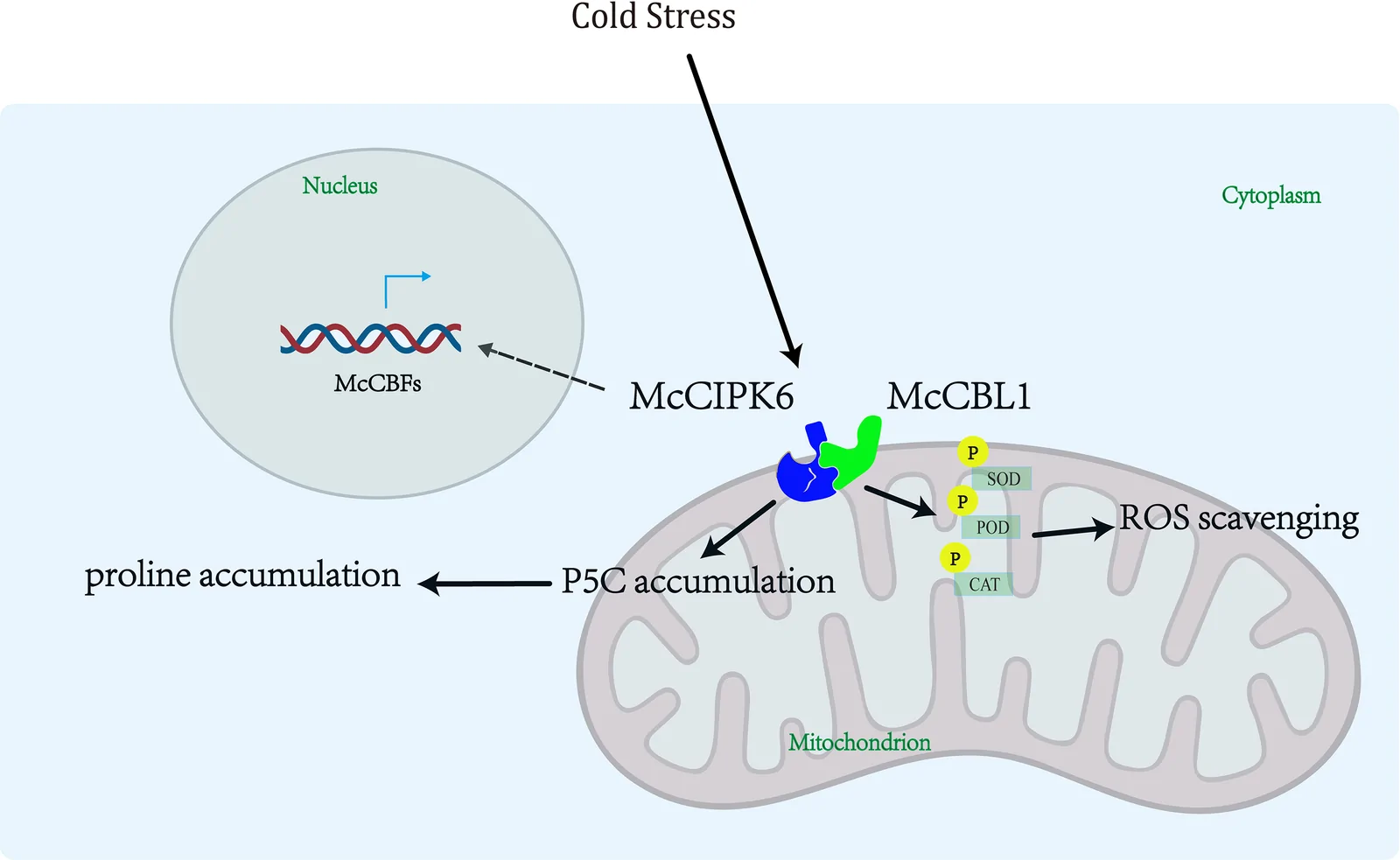
A proposed working model for McCIPK6-mediated cold stress tolerance in bitter gourd. Under cold stress, the mitochondrial-localized McCIPK6 is significantly induced and interacts with the calcium sensor McCBL1. McCIPK6 phosphorylates (P) the antioxidant enzymes SOD, POD, and CAT in mitochondria to enhance ROS scavenging capacity, thereby alleviating oxidative damage. Meanwhile, *McCIPK6* upregulates the expression of *McCBFs* in the nucleus to activate the *CBF* cold signaling pathway, and promotes P5C accumulation to facilitate proline biosynthesis, improving osmotic adjustment. Collectively, *McCIPK6* positively regulates cold tolerance in bitter gourd through the synergistic regulation of the ROS scavenging system and *CBF* signaling pathway, in a *McCBL1*-dependent manner.

In this study, we systematically explored the function and regulatory mechanism of *McCIPK6* in the response of bitter gourd to cold stress. Using cold-tolerant and cold-sensitive bitter gourd genotypes, we integrated molecular biology and protein–protein interaction analyses to elucidate the regulatory function of McCIPK6 in cold tolerance, aiming to provide a theoretical basis and candidate genes for cold-tolerance genetic improvement in bitter gourd. Our results demonstrated that the cold-tolerant genotype R maintained superior leaf structure and cellular integrity under cold stress and coped with cold stress by enhancing the activity of the antioxidant system and regulating the accumulation of osmotic substances. This is consistent with the universal cold resistance mechanism of plants reported in previous studies. Subcellular localization analysis showed that McCIPK6 was localized to mitochondria, and its expression was significantly induced by cold stress, indicating that McCIPK6 is closely involved in the early cold response of bitter gourd to cold stress. Heterologous overexpression of *McCIPK6* significantly improved cold tolerance in *Arabidopsis thaliana*, and McCIPK6 specifically interacted with McCBL1, thereby regulating cold tolerance by activating the *CBF* signaling pathway and enhancing ROS scavenging capacity. These findings clearly confirm that *McCIPK6* serves as a core positive regulator in the cold tolerance pathway of bitter gourd. Collectively, we propose a working model for *McCIPK6*-mediated cold tolerance in bitter gourd (**Figure 6**). Upon cold stress, the mitochondrial-localized McCIPK6 is rapidly and significantly induced and physically interacts with the calcium sensor McCBL1 to transduce the cold-induced Ca²^+^ signal. On one hand, McCIPK6 phosphorylates the antioxidant enzymes SOD, POD, and CAT in mitochondria, enhancing their activities to efficiently scavenge excessive ROS and alleviate cold-induced oxidative damage. On the other hand, *McCIPK6* upregulates the expression of *McCBFs* in the nucleus to activate the *CBF*-mediated cold signaling pathway and also promotes the accumulation of P5C to facilitate proline biosynthesis, thereby improving osmotic adjustment capacity under cold stress. Together, these two synergistic pathways confer enhanced cold tolerance in bitter gourd.

## 5 Conclusion

In summary, this study identified and functionally characterized the mitochondrial-localized cold-responsive gene *McCIPK6* from bitter gourd. Under low-temperature stress, *McCIPK6* exhibited rapid and distinct induction patterns in the cold-tolerant genotype ‘0208’ and cold-sensitive genotype ‘2206’. Heterologous overexpression of *McCIPK6* in Arabidopsis significantly enhanced cold tolerance. Protein interaction assays confirmed that McCIPK6 physically interacts with the calcium sensor McCBL1. These results demonstrate that *McCIPK6* serves as a positive regulator of cold tolerance in bitter gourd by coordinating the *CBF* pathway and ROS scavenging system through the CBL-CIPK module. This study elucidates a novel molecular mechanism underlying chilling tolerance in bitter gourd and offers a valuable candidate gene for genetic improvement of cold resistance in cucurbit crops.

## Data Availability

The datasets presented in this study can be found in online repositories. The names of the repository/repositories and accession number(s) can be found in the article/**Supplementary Material**.
