# Correction: Characterization of McCIPK6, a mitochondrial protein, positively regulating cold stress tolerance in bitter gourd (*Momordica charantia* L.)

**DOI:** 10.3389/fpls.2026.1973395

**Published:** 2026-09-02

**Authors:** Xuetong Yang, Kai Wang, Yuanyuan Xie, Feng Guan, Bo Shi, Xinjian Wan

**Affiliations:** 1 Institute of Vegetables and Flowers, Jiangxi Academy of Agricultural Sciences, Nanchang, China; 2 Jiangxi Key Laboratory of Horticultural Crops (Fruit, Vegetable & Tea) Breeding, Jiangxi Academy of Agricultural Sciences, Nanchang, China; 3 Jiangxi Engineering Research Center of Vegetable Molecular Breeding, Jiangxi Academy of Agricultural Sciences, Nanchang, China

**Keywords:** CBF signaling pathway, CIPK gene, cold stress, McCBL1, *Momordica charantia*, reactive oxygen species

In the published article, there was a mistake in the Funding statement. The grant number for the Natural Science Foundation of Jiangxi Province was displayed as “20244BCE52267”. The correct grant number is “20252BAC240105”.

The original version of this article has been updated.

