# Supplementary material for: Characterization of McCIPK6, a mitochondrial protein, positively regulating cold stress tolerance in bitter gourd (Momordica charantia L.)
Source: Front Plant Sci. 2026 May 21;17:1856408. doi: 10.3389/fpls.2026.1856408 (PMC13233347; doi:10.3389/fpls.2026.1856408)
Supplement: Supplementary file 1 [file Table1.docx]

**Table S1** List of primers and their uses.

| **Primer** | **Sequence** | **Used for** |
| --- | --- | --- |
| OE-*McCIPK6*-F  OE-*McCIPK6*-R | acacgctgacaagctgactctagcagatctATGGCGGAAAAGGATAGAGA  aaaagttcttctcctttgcccatggctctagaaTCAAGCCGGCATGGAGTTCT | Overexpression |
| S-*McCIPK6*-F  S-*McCIPK6*-R | caattggagctccaccgcggtggcggccgcATGGCGGAAAAGGATAGAGA  gctcctcgcccttgctcaccatggtaccAGCCGGCATGGAGTTCTC | Subcellular localization |
| G-*McCIPK6*-F  G-*McCIPK6*-R | tgactccatgtcgacggatccAATTTATTTTTATTTTTTTCAA  ggtggactcctcttagaattcCGATTCGATCTTCTTCTTTCA | GUS |
| pGBKT7-McCIPK6-F  pGBKT7-McCIPK6-R | catatggccatggaggccgaattcATGGCGGAAAAGGATAGAGA  ggccgctgcaggtcgacggatcccTCAAGCCGGCATGGAGTTCT | Y2H |
| pGADT7-McCBL1-F  pGADT7-McCBL1-R | gctcatatggccatggaggccagtgaattcATGGGCTGTATTCAGTCAAAA  ttcatctgcagctcgagctcgatggatcccTTATGTGGCAATCTCATCCAC |  |
| pCAMBIA1300-cLUC-McCIPK6-F  pCAMBIA1300-cLUC-McCIPK6-R | aagcagatctcgtacgcgtcccggggcggtacccggtacccATGGCGGAAAAGGATAGAGACTGT  gatacgaacgaaagctctgcaggtcgacTCAAGCCGGCATGGAGTTCTCCGT | LUC |
| pCAMBIA1300-nLUC-McCBL1-F  pCAMBIA1300-nLUC-McCBL1-R | atttggagagaacacgggggacgagctcgATGGGCTGTATTCAGTCAAAAGGA  gtcaggaacatcgtatgggtagtcgacTGTGGCAATCTCATCCACCTCAGA |  |
| q-*McCIPK6*-F  q-*McCIPK6*-R | \| ATGGCGGAAAAGGATAGAGA \| \| --- \| \| TCCTTCCCAACCACCTTCAT \| | qRT-PCR |
| q-*AtCBF1*-F  q-*AtCBF1*-R | TGTCTCAACTTCGCTGACTCGGC  ACCTTCGCTCTGTTCCGGTGTATAA |  |
| q-*AtCBF2*-F  q-*AtCBF2*-R | GGTTTCCTCAGGCGGTGATTACAGT  TCAGCGGTTTGGAAAGTCCCGAGCC |  |
| q-*AtCBF3*-F  q-*AtCBF3*-R | TATTTCAGCAAACCATACCAAC  CTCTAACCTCACAAACCCACTT |  |
| q-*AtPOD1*-F  q-*AtPOD1*-R | TCTGACCGTTCAAGAAATGG TGGAGCAACCCGTAACCGTG |  |
| q-*AtSOD*-F  q-*AtSOD*-R | ACTGCCACCTTCACAATCACTG GCTTTAGCCCTGGAGACCAATG |  |
| q-*AtCAT1*-F  q-*AtCAT1*-R | CGTGAAGCGTTTTGTTGAAGC  CGAGTTGCTAGTTTCTGTCCCAG |  |
| *McActin* 7-F  *McActin* 7-R | CCCTCCCTCATGCAATTCTC  GTTACATGTTTACCACTACTGCCGA |  |
| *AtActin*-F  *AtActin*-F | GCACCAAGCAGCATGAAGA  GAACCACCGATCCAGACACT |  |
